# Targeting *Clostridium difficile* Surface Components to Develop Immunotherapeutic Strategies Against *Clostridium difficile* Infection

**DOI:** 10.3389/fmicb.2018.01009

**Published:** 2018-05-23

**Authors:** Séverine Péchiné, Jean F. Bruxelle, Claire Janoir, Anne Collignon

**Affiliations:** EA 4043, Unités Bactéries Pathogènes et Santé, Université Paris-Sud, Université Paris-Saclay, Châtenay-Malabry, France

**Keywords:** *Clostridium difficile*, surface components, passive immunotherapies, vaccines, protection

## Abstract

New therapies are needed to prevent and treat *Clostridium difficile* infection and to limit the rise in antibiotic resistance. Besides toxins, several surface components have been characterized as colonization factors and have been shown as immunogenic. This review will focus on passive and active immunization strategies targeting *C. difficile* surface components to combat *C. difficile*. Concerning passive immunization, the first strategies used antisera raised against the entire bacterium to prevent infection in the hamster model. Then, surface components such as the flagellin and the S-layer proteins were used for immunization and the passive transfer of antibodies was protective in animal models. Passive immunotherapy with polyvalent immunoglobulins was used in humans and bovine immunoglobulin concentrates were evaluated in clinical trials. Concerning active immunization, vaccine assays targeting surface components were tested mainly in animal models, mouse models of colonization and hamster models of infection. Bacterial extracts, spore proteins and surface components of vegetative cells such as cell wall proteins, flagellar proteins, and polysaccharides were used as vaccine targets. Vaccine assays were performed by parenteral and mucosal routes of immunization. Both gave promising results and pave the way to development of new vaccines.

## Introduction

*Clostridium difficile* is involved in a wide spectrum of diseases from mild post-antibiotic diarrhea to pseudomembranous colitis (PMC) that can be severe and lead to complications such as toxic megacolon, septic shock, and death (Rupnik et al., 2009). *C. difficile* infections (CDI) are often nosocomial but can also be community-acquired. Contamination occurs with ingestion of spores, which germinate in the intestinal tract allowing vegetative cells to colonize the gut and multiply in the colon. Several studies showed that several surface components have a role in the colonization step. Colonization is favored by dysbiosis of the intestinal microbiota often due to antibiotic treatment. Then, the toxins TcdA and TcdB are released and are responsible for the intestinal symptoms. The treatment of a first episode is based on specific antibiotic treatment. However, a major problem of these infections is the high level of recurrences 20–30% after a first treatment of an initial infection and more than 50% after a first recurrence (Shields et al., 2015). CDI is an important healthcare-associated disease, causing almost 500,000 infections each year in the United States. *C. difficile* infections have a significant cost on healthcare systems (Lessa et al., 2015). The United States Center for Disease Control has designated this pathogen as the pre-eminent of three “Urgent Threats” to United States healthcare. Besides first line antibiotic treatments, which target *C. difficile* but also species of the normal microbiota, new therapeutic strategies are needed to treat CDI and prevent recurrences. These new strategies must preserve the normal intestinal microbiota and its barrier effect and limit rise in antibiotic resistance.

A better knowledge of *C. difficile*-host interaction has highlighted the numerous risk factors of CDI. Susceptibility to CDI is well-known and depends on host exposure to *C. difficile*, treatments facilitating *C. difficile* host colonization and a wide range of host characteristics. Host risk factors are age greater than 65 years, co-morbidities such as chronic diseases and host immune response. Several studies have reviewed the role of innate and adaptive immunity in CDI outcome (Madan and Petri, 2012; Péchiné and Collignon, 2016; Rees and Steiner, 2017; Vargas et al., 2017). For over 30 years, immunodeficiency and hematological malignancies such as neutropenia have been described as important risk factors for severe CDI (Rampling et al., 1985; Selvey et al., 2016). In the United States, between 2005 and 2011, a study on more than one million patients with leukemia showed that the susceptibility to CDI in this population was 2.6 times greater than non-leukemia patients (Luo et al., 2015). However, in humans it is difficult to pinpoint the role of the immune response in the susceptibility to CDI because of the accumulation of risk factors in immunodeficient patients such as multiple antibiotic treatments (Fisher et al., 2014).

Interestingly, several studies showed that the intensity of the specific host immune response to *C. difficile* exposure is a key factor in CDI outcome. Thus, the inability to develop a humoral immune response to *C. difficile* may be a prediction marker for severe and/or recurrent CDI (Kelly, 2012). In particular, anti-toxin antibody levels have been correlated with the severity, duration and recurrence rate of CDI (Warny et al., 1994; Kyne et al., 2000, 2001; Leav et al., 2010; Wullt et al., 2012). The first developed immunotherapies targeted toxins with the promising but recently stopped clinical development of CDIFFENSE vaccine by Sanofi Pasteur and with the FDA approved passive immunotherapy targeting TcdB, bezlotoxumab (Navalkele and Chopra, 2018). Although promising results in the decrease of CDI and recurrences, vaccines and passive immunotherapies targeting toxins do not appear to prevent *C. difficile* gut colonization, and further dissemination of spores in the environment. Therefore, another approach to prevent CDI and recurrences is to directly target the bacterium by developing immunotherapeutic strategies specific for *C. difficile* surface components.

In this review, first we introduce the characterized *C. difficile* surface components and the host specific immune response, then we evaluate the different passive and active immunization strategies targeting *C. difficile* surface components.

## Host Immune Response Against *C. difficile* Surface Components

Serum antibodies against *C. difficile* surface components have been found in patients with CDI (Pantosti et al., 1989; Mulligan et al., 1993). Pantosti et al. (1989) compared the antibody response in the acute and convalescent phase and observed that the IgG response increased during disease outcome. Mulligan et al. (1993) found that the level of immunoglobulins against somatic cell antigens was higher in asymptomatic than in symptomatic carriers.

A better knowledge of *C. difficile* pathogenesis has shed light on several components involved in intestinal colonization.

### Surface Proteins of the Vegetative Cells

A major family of surface proteins is the cell wall protein (CWP) family (Willing et al., 2015), in which the S-layer proteins, the adhesin Cwp66, and the protease Cwp84 are implicated in the colonization step (Janoir, 2016).

The two S-layer proteins (SLPs) are the most abundant surface proteins and form a two-dimensional array around the bacterium. They derive from the S-layer precursor SlpA after cleavage by the Cwp84 protease (Kirby et al., 2009; Dang et al., 2010). The low-molecular-weight (LMW)-SLP is surface-exposed and highly variable between strains while the high-molecular-weight (HMW)-SLP is conserved between strains and anchored in the cell wall (Karjalainen et al., 2001). Both SLPs are capable of adhering *in vitro* to human gastro-intestinal tissue and intestinal epithelial cells (Calabi et al., 2002; Merrigan et al., 2013). The HMW-SLP also adheres to extra cellular matrix (ECM) proteins including type 1 collagen, thrombospondin and vitronectin (Calabi et al., 2002). In addition, SLPs interact with TLR 4 inducing a pro-inflammatory innate immune response of the host (Ryan et al., 2011). Then, SLPs induce an adaptive immune response. For a long time, it has been impossible to obtain SLP mutants, suggesting essentiality of the *slpA* gene. Recently, Kirk et al. (2017) described rare resistant mutants to a diffocin (Av-CD 291.2), which selectively kills *C. difficile* strains. Two of these resistant mutants displayed a SLP-null phenotype. These mutants displayed severe sporulation defects, and were capable of colonizing the intestinal tract of hamsters despite a complete attenuation of virulence in this animal model (Kirk et al., 2017).

Wright et al. (2008) by an immuno-proteomic-based approach demonstrated that the SLPs are detected by patient sera suggesting that these proteins are immunodominant and expressed during infection (Wright et al., 2008). Drudy et al. (2004) found that antibody response to SLPs (total extracts of both SLPs) was not significantly different between patients with symptomatic CDI, asymptomatic *C. difficile* carriers and healthy controls. However, “patients with recurrences compared to patients with a single episode of CDI did not mount a SLP-specific IgM immune response on days 3, 6, and 9 after onset of diarrhea” (Drudy et al., 2004). Recently, Bruxelle et al. assessed the immune response of the host against the SlpA precursor. In humans SlpA was immunogenic and patients with CDI had a significantly higher level of anti-SlpA antibodies as compared to healthy patients (Bruxelle et al., 2016). Interestingly, Negm et al. (2015) developed a microarray assay to screen antibody responses simultaneously against various *C. difficile* antigens from different *C. difficile* strains. By this assay the authors tested IgG antibodies against native or recombinant toxins and SLP extracts from various *C. difficile* strains in a CDI patient group, a group of patients with cystic fibrosis and a healthy group of subjects. Patients with CDI had significantly lower anti-SLP IgG levels with all strains tested (ribotypes 001, 002, 027) compared to the two other groups. These different results could be explained by the diversity of subjects in CDI groups but also in healthy control groups for which previous exposure to *C. difficile* in the community may lead to production of specific anti-SLP antibodies. In addition, variation in these data could also be explained by differences in the methodologies for antibody characterization, especially regarding the antigens, SLP- extracts, LMW-SLP, HMW-SLP or the precursor SlpA.

The Cwp66 protein also displays adhesin properties and its expression is increased *in vitro* after heat-shock (Waligora et al., 2001). It has a two-domain structure with the C-terminal domain (C-ter) surface exposed and highly variable between strains whereas the N-terminal domain is conserved and anchored in the call wall.

The protease Cwp84 is surface exposed and conserved between strains (Janoir et al., 2007; Chapeton Montes et al., 2013). Besides cleavage of the SlpA precursor into the two mature SLPs, this protease is capable of degrading *in vitro* collagen, fibronectin or vitronectin. These proteolytic properties may *in vivo* facilitate bacterial spread (Janoir et al., 2007). However, a *cwp84* mutant is as virulent as the wild type strain in the hamster model suggesting that this protease has a limited function in the pathogenesis and can be substituted by another protease such as Cwp13 (Kirby et al., 2009).

The C-terminal domain of Cwp66 and the Cwp84 protease are immunogenic in humans. Péchiné et al. comparing total antibody levels in a CDI group and a control group observed that the mean level of antibodies against Cwp66 C-ter and Cwp84 was statistically lower in the CDI group. These results suggest that the antibodies can be protective against CDI (Péchiné et al., 2005a,b).

Other proteins are surface-exposed and implicated in the colonization step. The heat shock protein GroEL is released extracellularly after heat shock and capable of binding to epithelial cells (Hennequin et al., 2001). This protein could serve adhesin function in case of various *in vivo* shocks. After immunization of hamsters with a non-toxigenic *C. difficile* strain, GroEL has been identified by an immuno-proteomic approach as a key factor leading to production of protective antibodies (Péchiné et al., 2013). GroEL is highly conserved between *C. difficile* strains but also displays high homology with other bacterial species of the normal microbiota. Thus, these homologies limit the interest of GroEL as vaccine antigen. The lipoprotein CD630-0873 displays adhesive properties on Caco-2 cells. In addition, a knockout mutant adheres significantly less than the parental strain to Caco-2 cells confirming the role of the lipoprotein in adherence (Kovacs-Simon et al., 2014).

The fibronectin-binding protein Fbp68 binds to fibronectin (soluble and immobilized) and to intestinal fibronectin-pre-incubated cells. Fbp68 could interact with fibronectin in host tissues. It is highly conserved between strains and displays 38 and 39% identity, respectively with PavA of *Streptococcus pneumoniae* and Fbp54 of *Streptococcus pyogenes* (Hennequin et al., 2003; Barketi-Klai et al., 2011). Manganese binds to the N-terminal domain of Fbp68 inducing a change of conformation of the protein essential for fibronectin binding (Lin et al., 2011). Interestingly knockout mutants of the *fbp* gene adhered more *in vitro* to epithelial cells than the parental strain confirming that adherence is a multifactorial process. *In vivo*, in monoxenic mice caecal colonization by the mutant was reduced compared to the wild type and in dixenic mice its intestinal implantation was slower but caecal colonization was similar (Barketi-Klai et al., 2011). So the adhesin Fbp68 could participate to intestinal colonization. This protein is expressed during the course of infection and is immunogenic in humans (Péchiné et al., 2005a). The collagen-binding protein CbpA has an N-terminal collagen-binding domain and a C-terminal domain anchored to the cell wall. A recombinant protein expressing the N-terminal domain was able to bind to collagen *in vitro* and expression of CbpA in *Lactococcus lactis* led to enhanced adherence to collagen (Tulli et al., 2013). However, a *cbpA* knockout strain adhered similarly to collagen compared to the wild type isogenic strain. Similarly, in a dixenic mouse model of colonization, the mutant and the parental strain showed similar level of intestinal colonization (Janoir et al., 2013). CbpA by interacting with host ECM proteins after disruption of the tight junctions by the toxins could facilitate the pathogenic process. Two teams characterized a secreted-zinc metalloprotease ZmP1 (CD630-28300) capable of cleaving IgA2, fibrinogen or fibronectin (Cafardi et al., 2013; Hensbergen et al., 2014). According to c-di-GMP concentration (low concentration), this protease is produced and can limit adherence to host proteins.

### Surface Filamentous Structures: Flagella and Pili

Most *C. difficile* strains are motile, however in some strains the flagellar organization is peritrichous, and in others polar. In addition, a few strains such as the PCR-ribotype 078 strains are not motile since the F3 flagellar locus is absent. The flagellin FliC and the cap protein FliD bind *in vitro* to murine mucus. *In vivo*, it was shown that non-flagellated strains adhered significantly less than flagellated strains to mouse caeca. These first results are in favor of the role of flagella in intestinal colonization (Janoir, 2016).

More recently, other authors compared the adherence and colonization properties of parental motile strains to knockout *fliC* or *fliD* mutants (Baban et al., 2013). Surprisingly, the results were different between 630 strain and R20291 027 strain. In the 630 background, *fliC* and *fliD* mutants displayed increased adherence to intestinal epithelial cells compared to the parental strain (Baban et al., 2013). In contrast, in the R20291 background *fliC* and *fliD* mutants showed decreased adherence compared to the parental strain. These authors concluded that for “the 630 strain flagella and motility are not essential for adherence and colonization whereas for *C. difficile* R20291 flagella may play a more significant role in bacterial adherence than initial motility-driven colonization.” Taken together these results showed that the role of flagella in *C. difficile* pathogenesis is complex and different according to strains. In addition, flagella can modulate toxin production and stimulate innate immunity via flagellin-TLR5 interaction. *C. difficile* flagella and flagellin have been shown to interact with TLR5 in various human epithelial cells (Yoshino et al., 2013; Batah et al., 2016). This interaction leads to CCL20 and IL-8 production. In an *in vivo* mouse model, Batah et al. (2016) compared cecal inflammation after challenge with the R20291 027 wild type strain or with various flagellar and toxin mutants. Their results strongly suggest that the flagellin in combination with toxins is responsible for intestinal inflammation.

After stimulating innate immunity flagella also induce an adaptive immune response. Patients with CDI develop antibodies to FliC and FliD. Péchiné et al. (2005a) showed that the mean level of serum antibodies against FliC and FliD was statistically lower in a CDI patient group as compared to a control group. These results concerning flagellar proteins but also CWPs and Fbp68 suggest a protective role of antibodies targeting colonization factors.

Genes for type IV pili are present in *C. difficile* strains such as the 630 and 027 R20291 strains. Nine pilin or pilin-like protein genes were described in the *C. difficile* R20291 genome. The N-terminal hydrophobic regions of these pilins are relatively conserved but their C-termini are divergent (Maldarelli et al., 2014; Piepenbrink et al., 2015). This genetic variability probably facilitates immune evasion to the host immune response. In *C. difficile*, Type IV pili have been shown to be involved in bacterial auto-aggregation at high c-di-GMP concentrations (Bordeleau et al., 2015).

### Polysaccharides and Lipoteichoic Acid

Ganeshapillai et al. (2008) identified PS-I and PS-II, two *C. difficile* cell wall polysaccharides. PS-I is a polymer of branched pentasaccharide phosphate-repeats composed of rhamnose and glucose. PS-II is a polymer of hexasacharide phosphate-repeats composed of glucose, mannose, *N*-aceylgalactosamine. PS-II appears to be a common antigen of *C. difficile* conserved across the majority of *C. difficile* strains.

PS-I is expressed in low levels in bacterial cultures and consequently is difficult to obtain by extraction. For this reason, Martin et al. (2013) synthesized the pentasaccharide repeating unit of PS-I and oligosaccharide substructures using boosted chemical synthesis protocols and produced large amounts of well-defined PS-I related glycans. The immunogenicity of this PS-I glycan has been confirmed by specific humoral immune response analysis in stool and serum samples obtained from CDI patients.

PSII is the most abundant polysaccharide expressed by most *C. difficile* ribotypes. Danieli et al. (2011) synthesized the surface polysaccharide PS-II repeating unit. To be fully immunogenic, polysaccharides have to be coupled to carrier proteins. Adamo et al. (2012) explored a combination of chemical synthesis to identify a synthetic fragment that after conjugation to a carrier protein would be immunogenic. They demonstrated that the phosphate group was a key factor in synthetic glycans to mimic the native PSII polysaccharide. Both native PSII and a phosphorylated synthetic hexasaccharide repeating unit conjugated to the diphtheria toxoid variant CRM197 could elicit immunogenic responses in mice (Adamo et al., 2012). The synthetic PS-II hexasaccharide hapten was specifically recognized by IgA antibodies extracted from stools of patients with CDI. Oberli et al. (2011) used PS-II combined with CRM197 to immunize mice by subcutaneous route. The PS-II-CRM197 conjugate was immunogenic and induced specific IgG antibodies in the serum of immunized mice (Oberli et al., 2011). In another study, a non-adjuvanted PSI/PSII preparation was administered to pregnant sows as vaccine antigens and IgM antibodies specific for PSII were then elicited (Bertolo et al., 2012). Romano et al. (2014) evaluated in a mouse model the efficacy of PSII conjugated to recombinant toxins A (TcdA-B2) and B (TcdB-GT) fragments as carriers. Both glycoconjugates induced anti-PSII IgG but TcdB-GT conjugate was the most potent (Romano et al., 2014).

Reid et al. (2012) identified in all *C. difficile* strains tested, a carbohydrate polymer with the structure of a lipoteichoic acid (LTA) also called PS-III. Cox et al. (2013) examined LTA as a vaccine candidate. Two carrier proteins were used either human serum albumine (HSA) or a genetically inactivated *Pseudomonas aeruginosa* exotoxin A (ExoA). The attachment point for conjugation was an amino group present at the *N*-acetyl-glucosamine residues within the LTA polymer-repeating unit. These conjugates were used to immunize by parenteral route rabbits and mice. Immune sera recognized live vegetative cells and spore forms in an immunofluorescence assay, confirming that the LTA polymer is a highly conserved surface polymer of *C. difficile* inducing a specific immune response. The immunogenicity of ExoA-PS-III conjugate was better than that of the HSA-PS-III conjugate. However, the immune sera recognized all strains of *C. difficile* tested and also *C. butyricum*, *C. subterminale*, and *C. bifermentans* but not *C. perfringens*, *C. sporogenes*, *C. barati*, and *C. botulinum* (Cox et al., 2013).

### Surface Proteins of the Spore

The first step of pathogenenesis is the ingestion of spores due to contamination. After contamination by spores, germination begins in the intestinal tract. Spores are highly resistant and responsible for persistence and dissemination. The spore envelope comprises the peptidoglycan cortex, a coat composed of structural and enzymatic proteins and the exosporium predominantly composed of proteins such as BclA glycoproteins and Cde cystein-rich proteins (Paredes-Sabja et al., 2014).

## Passive Immunizations Targeting *C. difficile* Surface Components

The role of the antibody response in CDI outcome has been emphasized particularly for antibodies targeting toxins but also the whole bacterial cells (Kyne et al., 2001). Various passive immunization strategies have been tested to prevent or cure CDI and prevent recurrences. While most of these strategies target the toxins TcdA and TcdB and consequently neutralize the toxin activity, others target the whole bacterial cell or specific surface components (**Table 1**).

**Table 1:** Passive immunization strategies targeting *C. difficile* surface components.

Antigen	Antibody	Route of administration	Schedule	Model	Outcome	Reference
TcdA and TcdB + whole bacterium	Immune whey protein concentrate (WPC-40, Mucomilk)	Oral	Before and after challenge, then every 8 h during 10 days	Hamster	80–90% protection	van Dissel et al., 2005
			Three times daily for 2 weeks after antibiotic treatment	CDI patients	Significant decrease of recurrences	Numan et al., 2007
Formalin inactivated *C. difficile* cells	Immune whey IgG concentrate (CDIW)	Oral	Three times daily, 14 days	Randomized double-blind study in CDI patients	As effective as metronidazole in the prevention of recurrences	Mattila et al., 2008
TcdB-C-ter, inactivated spores, exosporium, inactivated vegetative cells, SLPs	Hyper-immune bovine colostrum TcdB-HBC, Mix1-HBC, Mix2-HBC	Oral	Two days before challenge and throughout experiment	Mouse model of infection and relapse	HBC-TcdB alone or in combination (Mix1 and Mix2-HBC) prevents and treats CDI in mice and reduces recurrences	Hutton et al., 2017
LMW- and HMW-SLPs	Rabbit hyper- immune serum	Oral	Seven hour before challenge, during challenge, then 6, 17, and 24 h after challenge	Hamster	Prolonged survival after challenge but no protection against death	O’Brien et al., 2005
FliC	Mouse hyper- immune serum	Intra-peritoneal	Twenty four hour before challenge	Mouse	Eighty percent of protection	Ghose et al., 2016b

### Human Polyvalent Immunoglobulins

First, intravenous (i.v.) immunoglobulin therapy has been used in cases of severe and recurrent CDI in infants and adults simultaneously or after antibiotic standard of care treatment. These normal immunoglobulins may contain antibodies against toxins, which after leakage through the inflamed intestinal mucosa may neutralize the toxins. Although intra-venous gamma globulins (IVGG) have been tested in many patients with CDI, the results are varied and make recommendation for use difficult (O’Horo and Safdar, 2009; Diraviyam et al., 2016). Negm et al. (2017) observed differences in TcdA neutralizing efficacy between three commercial immunoglobulin preparations as well as differences in level of specific IgG isotypes against *C. difficile* antigens.

### Bovine Antibodies

After the use of polyvalent human immunoglobulins, animals were immunized with *C. difficile* filtrates or whole extracts. Lyerly et al. (1991) orally vaccinated gestating cows with *C. difficile* formalin inactivated culture filtrate and obtained a bovine immunoglobulin G (IgG) concentrate (BIC) to orally passively immunized hamsters. After *C. difficile* challenge, immunized hamsters were protected from disease during BIC administration but died after treatment cessation. These BICs contained neutralizing IgG against toxins and probably to other antigens. These results were the first to demonstrate that passive oral immunization can protect partially against *C. difficile* lethal infection.

The main interest of colostrum antibodies is their stability in the digestive tract (Kelly et al., 1997). Later, van Dissel et al. (2005) immunized cows with killed whole bacterial cells and the two inactivated toxins TcdA and TcdB. They collected the milk of immunized cows to produce an immune whey protein concentrate (Immune WPC-40; Mucomilk). This concentrate contained secretory IgA (sIgA) antibodies recognizing the whole bacterium and the two toxins (van Dissel et al., 2005). *In vitro*, this WPC was able to neutralize the cytotoxicity of toxins. *In vivo*, in the lethal hamster model challenged with the toxigenic *C. difficile* VPI 10463 strain, oral administration by gavage of 1 mL of WPC 3 h before and after *C. difficile* challenge followed by administration every 8 h for 3 days conferred 80–90% protection of hamsters. In contrast to Lyerly et al. (1991), hamsters survived for at least 28 days after the end of treatment. These authors suggest that specific sIgA against the whole bacterial cell may decrease *C. difficile* gut colonization. Then van Dissel et al. (2005) tested WPC-40 as a medical food in humans to prevent relapses. In an uncontrolled cohort study, WPC-40 was tested in 16 patients with CDI, 9 of whom had one or more relapses. After a standard antibiotic treatment, WPC-40 was given three times daily for 2 weeks. For each dose, 5 g of WPC-40 was diluted in flat mineral water and administered orally before each meal. This treatment was well-tolerated without adverse effects. After treatment, no toxin was detected in fecal samples in 14 out of 15 patients and *C. difficile* was no longer detected in stool culture in 9 out of 15 patients. None of the patients experienced another episode of CDI after treatment during the follow-up period (median 333 days). To confirm this first human study, WPC-40 was tested in a larger cohort of 101 patients with CDI (median age 74 years). After at least 10 days of antibiotic treatment, WPC-40 was given orally for 2 weeks. As previously the daily dose was 15 g of WPC. Interestingly, only 10% of patients relapsed within the 60 days follow-up (Numan et al., 2007). The authors concluded that oral administration of WPC-40 might help in the prevention of relapses. A phase 2 clinical trial has been performed but the results are not yet posted (NCT00177775).

Another team from Finland also produced an immune whey IgG concentrate (CDIW) using the colostrum of cows immunized with formalin inactivated *C. difficile* (Mattila et al., 2008). In a prospective, randomized, double blind study, they compared CDIW (200 ml three times daily) with metronidazole (400 mg three times daily) to prevent relapses in patients with at least two previous episodes of CDI. After 14 days of treatment, metronidazole was effective in 100% of patients (20) compared to 89% (16/18) for CDIW. At the end of the follow-up study (70 days), the number of patients with relapse was not statistically different between the two groups (44% in CDIW treated group, 45% in metronidazole group). The authors concluded that in this preliminary study CDIW was as effective as metronidazole in the prevention of CDI recurrences and was well-tolerated. Unfortunately, the study was early interrupted.

Recently an Australian team developed hyper-immune bovine colostrum (HBC) to prevent and treat CDI (Hutton et al., 2017). These authors used different *C. difficile* components (027 strain DLL3109) to immunize pregnant cows: the TcdB-C-terminal binding domain, inactivated whole spores or an exosporium extract, and inactivated vegetative cells or a SLP-preparation. They obtained different HBC, named TcdB-HBC, Spore-HBC, Exo-HBC, Veg-HBC, and SLP-HBC respectively. In a mouse model of CDI infections (primary infection and relapse), these HBCs were able to prevent and treat CDI. For prophylactic administration HBC was given *ad libidum* 2 days prior infection and throughout the experiment. Mice treated with Veg-HBC or SLP-HBC did not survive infection. In contrast, survival rates were significantly higher in Spore-HBC (*p* < 0.0001), Exo-HBC (*p* = 0.0075), TcdB-HBC (*p* < 0.0001), and vancomycin (*p* < 0.0001) treated mice compared to untreated mice, which died rapidly after *C. difficile* challenge. The survival rate was the highest in vancomycin and TcdB-HBC (70%) treated mice with no significant difference. Then, various mixtures of HBC in equal ratios were tested: Mix1-HBC contained Spore-HBC, Veg-HBC and TcdB-HBC, Mix2-HBC contained Exo-HBC, SLP-HBC, and TcdB-HBC and Mix3-HBC that contained TcdB-HBC diluted 1:3 in non-immune colostrum to correspond to the amount of TcdB-HBC present in Mix1 and 2. Interestingly, Mix1-HBC and Mix2-HBC treated mice had 70 and 80% survival rate respectively, whereas Mix3-treated mice died after 2 days as did control mice without treatment. Survival rates were not significantly different between vancomycin, TcdB-HBC, Mix1-HBC, and Mix2HBC treated mice. Spore enumeration was similar in all groups (10^6^ CFU/gram), indicating that these treatments had no effect on colonization. Mix2-HBC was also tested in a mouse disease relapse model. Control mice receiving no colostrum after vancomycin treatment had a low survival rate (11%). In contrast, colostrum-treated mice had a higher survival rate (77%) but always shedded spores in the feces suggesting no effect of this mixture on colonization. These authors concluded “that administration of HBC-TcdB alone or in combination with spore or vegetative cell-targeted colostrum prevents and treats CDI in mice and reduces recurrence.”

One of the main advantages of HBC is the oral administration, which offers several advantages as compared to parenteral administration such as easy administration, local activity in the intestinal tract, and lower cost of production.

Taken together these results suggest that it could be interesting to raise antibodies specific to surface components of the vegetative cell or the spore to unravel the role of specific-surface component antibodies in preventing intestinal colonization.

### Specific Antibodies to *C. difficile* Surface Components

#### Anti SLP Antibodies

Several studies suggest that the capacity to induce an immune response against SLPs may influence CDI outcome. O’Brien et al. (2005) produced rabbit polyclonal antibodies against SLPs. The SLPs used for immunization were purified from a crude SLP-extract from UK reference strain R13537 (ribotype 1). These antibodies reacted strongly with both LMW- and HMW-SLPs. Then, they tested these antibodies to prevent CDI in the hamster lethal model. Rabbit antiserum was administered in carbonate buffer pH 9.6 to neutralize gastric acid. 100 μl doses were given oro-gastrically to hamster 7 h before challenge, during challenge as a mixture pre-incubated with the *C. difficile* challenge inoculum, then 6, 17, and 24 h of infection. “This passive immunization strategy was unable to delay the onset of clinical symptoms and prevent death” but prolonged survival in *C. difficile* infected hamsters compared to untreated infected hamsters (medium post-challenge survival 156 h compared to controls 75 and 69 h). In addition, the authors demonstrated by an *in vitro* assay that anti-SLP antibodies enhanced *C. difficile* phagocytosis by monocytes. This partial protection could be explained by an inadequate level of antibodies (mainly IgG in this antiserum) reaching the intestinal mucosa.

Single-domain antibodies (VHHs or nanobodies) obtained from the variable domains of *Camelidae* heavy-chain IgGs have the affinity and specificity of corresponding monoclonal antibodies. In addition, they are resistant to extreme pH and proteases and are interesting candidates for oral administration (Péchiné et al., 2017). Kandalaft et al. (2015) produced VHHs targeting the SLPs from *C. difficile* strain 027 QCD-32g58. Specific VHHs were selected from an immune llama VHH phage display library (Kandalaft et al., 2015). VHHs bound QCD-32g58 SLPs with high affinity but also SLPs from other strains of various ribotypes. A few of them were resistant to pepsin at physiological concentration. A combination of three LMW-SLP specific VHHs inhibited *in vitro* motility.

#### Anti-flagellin Antibodies

Ghose et al. (2016b) immunized mice with *C. difficile* flagellin FliC (strain VPI 10463) and obtained specific hyper-immune serum. In a mouse model of CDI, 400 μl of FliC specific hyper-immune serum was intraperitoneally (i.p.) administered simultaneously with clindamycin administration. Then, 24 h later, mice were challenged with a *C. difficile* strain (UK1). Passive immunization led to protection of 80% of treated mice (four out five) whereas four out five control mice treated with a non-immune serum died between 3 and 7 days post-challenge. In addition, anti-FliC antibodies were detected in four out of five mice from the challenge until day 10. Since FliC plays a multifactorial role in the pathogenesis, protection elicited by anti-FliC antibody may inhibit various steps of *C. difficile* pathogenesis.

## Vaccines Targeting *C. difficile* Surface Components

Different vaccine strategies to prevent or cure CDI and recurrences have also been developed and tested in animal models (**Table 2**).

**Table 2:** Vaccine strategies in animal models targeting *C. difficile* surface components.

Antigen	Adjuvant	Route of administration	Schedule	Model	Outcome	Reference
Spore proteins CdeC, CdeM	Alum	Intraperitoneal	Days 0, 14, 28	Mouse and hamster	Hundred percent protection in mice. Eighty percent of protection in hamsters	Ghose et al., 2016a
Cell wall extract	Cholera toxin	Rectal	Days 0, 15, 30	Hamster	Partial protection against lethal challenge	Péchiné et al., 2013
LMW- and HMW-SLPs	Alum	Intraperitoneal	Days 0, 21, 42	Hamster	No significant protection	Ni Eidhin et al., 2008
SlpA precursor	Cholera toxin	Rectal	Days 0, 15, 30	Mouse and hamster	Significant decrease of intestinal colonization at day 10 after challenge in mice. Partial but not lasting protection in hamsters	Bruxelle et al., 2016
Cwp84	Cholera toxin	Rectal	Days 0, 15, 31	Hamster	Significant increased survival (33%). Absence of colonization in surviving hamsters	Péchiné et al., 2011
Cwp84	Pectin beads	Oral	Days 0, 15, 32	Hamster	Forty percent of protection	Sandolo et al., 2011
FliC	Alum	Intraperitoneal	Days 0, 14, 28	Mouse and hamster	Eighty nine percent of protection in mice 43 to 64% of protection in a dose dependent manner in hamsters	Ghose et al., 2016b
GroEL	Cholera toxin	Nasal	Days 0, 7, 14, 28	Mouse	Significant decrease of intestinal colonization	Péchiné et al., 2013
LTA-CRM197	Alum	Subcutaneous	Days 0, 15, 30	Mouse	Significant decrease of intestinal colonization	Broecker et al., 2016
Pilin	Freund’s adjuvant	Subcutaneous	Three immunizations	Mouse	Low antibody response. No protection	Maldarelli et al., 2016

### Spore Components as Vaccine Antigens

*Clostridium difficile* germinates, colonizes and persists in the human gut partly due to spore proteins. Ghose et al. (2016a) used two surface-bound spore proteins, CdeC and CdeM as vaccine candidates against *C. difficile*. The recombinant spore proteins administered i.p. with adjuvant (alum) were immunogenic in mice. Vaccination with high doses of CdeC and CdeM were able to protect mice (100% of protection) against CDI after challenge with *C. difficile* UK1. Vaccine assays in golden Syrian hamsters with the same spore proteins induced a protection of 80% against challenge with *C. difficile* 630Δerm strain (Ghose et al., 2016a). Vaccinated hamsters had serum IgG antibodies against CdeC and CdeM that have to reach the intestinal lumen to neutralize the spore proteins.

### Crude Extracts as Vaccine Antigens

Colonization is a crucial step in CDI. Several *C. difficile* surface proteins may contribute to colonization (Deneve et al., 2009) and could be used as vaccine antigens. It was shown many years ago that besides toxins, somatic antigens, and surface components induce an immune response in the host important during the course of infection (Pantosti et al., 1989; Mulligan et al., 1993).

Membrane fraction from non-toxigenic *C. difficile* was used as vaccine candidate. Mice subcutaneously vaccinated with a non-toxigenic *C. difficile* membrane fraction and TiterMax Gold adjuvant, produced serum IgG and intestinal fluid IgA specific to the antigen that were able to inhibit *in vitro* adherence of *C. difficile* to Caco-2 cells (Senoh et al., 2015). In addition, *C. difficile* surface proteins have been evaluated as vaccine antigens in the hamster model to prevent intestinal colonization. A cell wall extract of a non-toxigenic *C. difficile* strain administered intra-rectally with cholera toxin as adjuvant partially prevented death in hamsters after a *C. difficile* challenge (Péchiné et al., 2013).

### Cell Wall Proteins as Vaccine Antigens

Ni Eidhin et al. (2008) immunized hamsters i.p. and/or intranasally with LMW and HMW-SLP (crude SLP purified by anion exchange chromatography). Different adjuvants were used: alum, Ribi, cholera toxin, and chitosan glutamate (Ni Eidhin et al., 2008). Immunizations with alum as adjuvant led to an increase of serum anti-SLP IgG. However, anti-SLP IgG titers did not correlate with post-challenge survival. In addition, the median survival time was not significantly different between the immunized and control groups. Bruxelle et al. (2016) assessed the protective effect of vaccination using the recombinant SlpA precursor from the toxigenic *C. difficile* strain 630 on colonization and survival in mouse and hamster models. Immunization assays were performed by rectal administration of SlpA with cholera toxin as adjuvant. In both models, immunizations induced production of serum specific IgG and IgA. In the mouse model, immunizations elicited production of anti-SlpA sIgA and intestinal *C. difficile* colonization was significantly lower in immunized mice compared to control mice 10 days after challenge. In the hamster model, specific SlpA antibodies conferred a partial but not lasting protection against CDI (Bruxelle et al., 2016). More recently, immunizations have been performed by rectal route in a mouse model of CDI with SlpA associated to FliC as adjuvant. Ten days after *C. difficile* challenge, *C. difficile* intestinal colonization decreased significantly in mice immunized with SlpA and FliC as adjuvant compared to the control group (Bruxelle et al., 2017).

The cysteine protease Cwp84 could have an important role in the physiopathology of *C. difficile* (Péchiné et al., 2005b). In the hamster model of CDI, the protease Cwp84 was assessed as vaccine antigen administered by rectal route with cholera toxin as adjuvant. In the Cwp84 immunized groups, survival was significantly prolonged compared to the control group and enumeration of *C. difficile* in feces showed that surviving animals were not colonized by *C. difficile* (Péchiné et al., 2011). Thereafter, Sandolo et al. (2011) evaluated this Cwp84 protease encapsulated into pectin beads as an oral vaccine candidate in the hamster model. Pectin is non-toxic, resistant to gastric or intestinal enzymes and almost totally degraded by pectinolytic enzymes produced by the colonic microbiota. Three immunizations by the intragastric route were performed with beads encapsulating Cwp84 and compared with a control group receiving unloaded beads. Two days after challenge with spores of a toxigenic strain of *C. difficile*, all the control hamsters died. In contrast, 40% of hamsters vaccinated with Cwp84-loaded beads survived 10 days after challenge, proving that oral vaccination with Cwp84 leads to partial protection (Sandolo et al., 2011).

### Flagellar Proteins as Vaccine Antigens

After passive immunization of mice using anti-FliC polyclonal serum confirmed protection to be antibody-mediated, Ghose et al. (2016b) performed vaccine assays by the parenteral route in mice and hamsters. Administered i.p. with alum as adjuvant, FliC afforded partial protection (around 50%) against CDI and death in hamsters challenged with *C. difficile* 630Δerm. Interestingly, these immunizations had no adverse effect on the digestive microbiota. In mice i.p. immunized with recombinant FliC, “the protection from CDI (UK1 *C. difficile* strain) and death correlated with a dose response and the number of immunizations received” (Ghose et al., 2016b). The highest protective efficacy of 89% (eight out nine mice disease free) was obtained with three doses of 25 μg of FliC. Notably, immunized mice shed significantly lower levels of spores as compared to non-immunized mice.

Recently, Potocki et al. (2017) combined approaches “to adsorb and display the *C. difficile* flagellar cap protein FliD on the surface of recombinant IL-2-presenting spores of *Bacillus subtilis.”* Intranasal immunizations of mice led to the development of a FliD-specific immune response. However, no protection assays in animal models have been performed with this strategy (Potocki et al., 2017).

### GroEl as Vaccine Antigen

Among cell surface proteins of *C. difficile*, the heat-shock protein GroEl has been tested as vaccine candidate for immunization against CDI. Intranasal immunization of mice with the recombinant protein GroEL and cholera toxin as adjuvant led to a significant decrease of *C. difficile* intestinal colonization after challenge compared to the control group (Péchiné et al., 2013).

### Polysaccharides and Lipoteichoic Acid as Vaccine Antigens

Different vaccine candidates have been evaluated in the mouse model for their immunogenicity. Firstly, a synthetic PS-I repeating unit conjugated to the diphtheria toxoid variant CRM197 was immunogenic in mice and induced immunoglobulin class switching. Second, the disaccharide Rha-(1→3)-Glc has been identified by microarray screening as a minimal epitope. Therefore, a CRM197-Rha-(1→3)-Glc disaccharide conjugate was evaluated. It was able to elicit antibodies recognizing the *C. difficile* PS-I pentasaccharide. These authors concluded “that the synthetic PS-I pentasaccharide repeating unit as well as the Rha-(1→3)-Glc disaccharide could be promising vaccine candidates against *C. difficile*” (Martin et al., 2013).

A parenteral vaccine composed of PS-II conjugated to keyhole lympet hemocyanin (KLH) led to the protection of 90% of mice after challenge with *C. difficile* spores (Monteiro, 2016).

LTA was identified as a possible vaccine antigen to prevent colonization. Broecker et al. (2016) reported on the potential of synthetic LTA glycans as vaccine candidates. They identified LTA-specific antibodies in the blood of *C. difficile* patients. Then, they evaluated the immunogenicity of a semi-synthetic LTA-CRM197 glycoconjugate. Mice were immunized subcutaneously three times with the conjugate associated to alum as adjuvant. This conjugate elicited LTA-specific antibodies in mice that recognized natural LTA epitopes on the surface of *C. difficile*. After oral challenge with *C. difficile*, the degree of bacterial colonization was significantly reduced as compared to control groups (Broecker et al., 2016).

### Pili as Vaccine Antigens

Maldarelli et al. (2014) described nine pilin or pilin-like protein genes. Six proteins were purified and used to immunize mice. “Immunizations of mice led to antibody responses that varied in titer and cross-reactivity, a notable result given the low amino acid sequence identity among the pilins” (Maldarelli et al., 2014). More recently these authors immunized mice with various pilins, whether combined or as individual proteins. Unfortunately, “low anti-pilin antibody titers and no protection upon *C. difficile* challenge were observed” (Maldarelli et al., 2016).

## Conclusion

The two main strategies in the development of specific immunotherapeutics against *C. difficile* target either the toxins or surface components. While neutralizing toxins can prevent clinical signs, an immunotherapy targeting surface components aims to prevent the first step of *C. difficile* pathogenesis gut colonization. Passive immunization with BIC has shown promising results in preventing recurrences in humans. A main advantage of BIC is the oral route of administration that allows delivery of antibodies directly in the colon to neutralize *C. difficile*. Given the multifactorial aspect of *C. difficile* pathogenesis, another strategy to broaden the protection is to target both toxins and colonization factors. Passive immunotherapy has the advantage of rapidity of action, which is essential for treating severe CDI. In contrast, the half-life of antibodies is short and the length of protection is restricted to a period of time. One advantage of vaccination is to develop long-term protection for patients at risk of CDI. Several assays of vaccine targeting surface components of *C. difficile* vegetative cells and spores have been performed in animal models. Some have given interesting results of protection against primary or recurrent CDI but most of the time protection was only partial. Combining several *C. difficile* surface components as vaccine antigens has the advantage of inhibiting several colonization factors involved in the colonization process and consequently could lead to an improved protection. The immune response against *C. difficile* has to be locally effective in the intestinal mucosa, hence the interest of developing vaccine by the mucosal route. Different antigen delivery systems and adjuvants have been tested in animal models to vaccinate by mucosal route against *C. difficile*. To date, only one strategy tested in phase 1 clinical trial aims to elicit both mucosal and systemic immune response to *C. difficile.* This vaccine is a combination of toxoid antigen and a spore component expressed on the surface of inactivated *Bacillus subtilis* spores (CDVAX) (NCT02991417, no result posted).

To conclude, strong scientific evidence supports the development of immunotherapies against *C. difficile* to prevent primary and recurrent CDI and to reduce gut colonization. However, the development of an effective vaccine against *C. difficile* faces many challenges. A key factor is the choice of the antigen. Targeting a panel of antigens could broaden the neutralization of *C. difficile* virulence factors and lead to potent therapeutic efficacy.

## Author Contributions

AC and CJ wrote the paragraphs on surface components and passive immunization strategies. SP and JB wrote the paragraph on vaccines. All the authors were involved in concept and design of the manuscript and reviewed and edited the manuscript.

## Conflict of Interest Statement

The authors declare that the research was conducted in the absence of any commercial or financial relationships that could be construed as a potential conflict of interest.
